# Directional freezing for the cryopreservation of adherent mammalian cells on a substrate

**DOI:** 10.1371/journal.pone.0192265

**Published:** 2018-02-15

**Authors:** Liat Bahari, Amir Bein, Victor Yashunsky, Ido Braslavsky

**Affiliations:** The Hebrew University of Jerusalem, Institute of Biochemistry, Food Science and Nutrition, The Robert H. Smith Faculty of Agriculture, Food and Environment, Rehovot, Israel; University of California at Berkeley, UNITED STATES

## Abstract

Successfully cryopreserving cells adhered to a substrate would facilitate the growth of a vital confluent cell culture after thawing while dramatically shortening the post-thaw culturing time. Herein we propose a controlled slow cooling method combining initial directional freezing followed by gradual cooling down to -80°C for robust preservation of cell monolayers adherent to a substrate. Using computer controlled cryostages we examined the effect of cooling rates and dimethylsulfoxide (DMSO) concentration on cell survival and established an optimal cryopreservation protocol. Experimental results show the highest post-thawing viability for directional ice growth at a speed of 30 μm/sec (equivalent to freezing rate of 3.8°C/min), followed by gradual cooling of the sample with decreasing rate of 0.5°C/min. Efficient cryopreservation of three widely used epithelial cell lines: IEC-18, HeLa, and Caco-2, provides proof-of-concept support for this new freezing protocol applied to adherent cells. This method is highly reproducible, significantly increases the post-thaw cell viability and can be readily applied for cryopreservation of cellular cultures in microfluidic devices.

## Introduction

Cell culture methods are routinely used in many fields and are indispensable for a variety of applications in basic research, clinical practice, medical diagnostics, and the pharmaceutical industry. Cell culturing is a labor-intensive and time-consuming process that involves multiple manipulations. Cryopreserving cells is an important part of the culturing process and is needed to preserve the original cellular characteristics during cell storage over long starches of time. For that, cryopreservation methods must provide significant survival rates and normal cell functionality after thawing for a wide range of cell types.

Cells are most commonly cryopreserved while dispersed in specialized freezing solutions. Preservation protocols involve detaching adherent cells from a substrate using a proteolytic enzyme (e.g., trypsin) and adding ‘cryoprotective agents’ (CPAs). This step is followed by a slow freezing protocol (1°C/min) and storage at -80°C or -196°C. While thawing is typically a rapid process (< 2 minute), the following steps needed for preparation of cell cultures for experiments can take days or weeks [1], depending on the cell proliferation rate and other biological processes such as differentiation. In some cases, cell recovery after preservation is especially challenging and time-consuming, for example, among slowly proliferating cells (e.g., embryonic stem cells [2]) and complex cell networks (e.g., neuronal networks [3] or polarized epithelial cells [4]). Furthermore, CPAs, such as dimethylsulfoxide (DMSO), ethylene glycol, and glycerol, are toxic to cells [5] especially at high concentrations.

Cryopreserving cells in an adherent state can significantly shorten and simplify the post thawing culturing steps. Furthermore, this approach unveils new opportunities for specific *in vitro* cell-based assays. For example, cryopreservation of adherent cells can facilitate direct storage in microfluidic devices (e.g. organs-on-a-chip), or the preservation of small numbers of cells.

As in the cryopreservation of dispersed cells, the cryopreservation of adhered cells faces challenges associated with avoiding cell injury, particularly within intermediate freezing temperatures (-15°C to -60°C) [6, 7]. Intracellular ice formation and osmotic injury are the main mechanisms leading to cell damage when freezing and thawing [7]. Additionally in the case of adhered cells, adjacent cells share cell–cell junctions, such as gap junctions, which allow solution transport between cells and can serve as a path for ice growth leading to sequential freezing [8–11].

Several studies have demonstrated the feasibility of cryopreserving cells adhered to a substrate [3, 12–22] exploring the effects of CPAs composition, extracellular matrix design, and substrate modification. However, no in depth study has been reported on optimization of the cryopreservation procedure of adherent cultures in terms of cooling rates and ice geometry.

While directional freezing was previously shown to support survival of suspended cells [23–26] and organs [27, 28], it was never utilized to the best of our knowledge for cryopreservation of adherent cell cultures. Here we studied the effect of directional freezing followed by gradual cooling on survival rates of adherent epithelial cell cultures (IEC-18, HeLa, and Caco-2). Specifically, we tested the influences of the ice growth rate in the course of directional freezing and cooling rates during the gradual cooling phase on cell survival. Additionally, we studied the impact of lowering the DMSO concentrations on post-thaw cell viability.

## Materials and methods

### Cell culture

Human intestinal Caco-2 cells (purchased from the American Type Culture Collection–ATCC) were cultured in Dulbecco’s modified Eagle’s medium (DMEM; Sigma-Aldrich, Inc., St. Louis, MO, USA) supplemented with 20% (v/v) fetal bovine serum (FBS; Biological Industries, Beit Haemek, Israel) and 1% (v/v) penicillin-streptomycin solution (Biological Industries). The human cervical cancer HeLa cell line (courtesy of Prof. Nahum Shpigel, The Hebrew University of Jerusalem, Israel) was cultured in DMEM medium (Sigma-Aldrich) supplemented with 10% (v/v) FBS (Biological Industries) and 1% (v/v) penicillin-streptomycin solution (Biological Industries). The rat intestinal epithelium IEC-18 cell line (courtesy of Prof. Betty Schwartz, The Hebrew University of Jerusalem, Israel) was cultured in DMEM (Sigma-Aldrich) supplemented with 10% (v/v) FBS (SAFC Biosciences Lenexa, KS, USA), 1% (v/v) L-glutamine (Biological Industries, Beit Haemek, Israel), and 1% (v/v) penicillin-streptomycin solution (Biological Industries). All cells were grown under a 37°C humidified atmosphere containing 95% air and 5% CO_2_.

### Freezing setups

Two cooling setups were developed to control the freezing of cell monolayers adhered to thin (170 to 250 μm) glass coverslips 10 to 20 mm in diameter (for round coverslips) or width (for rectangular coverslips). The first setup involved a translational cryostage that permitted motion-controlled directional freezing and simultaneous microscopic imaging. The second setup involved a liquid nitrogen (LN) flow cooling stage that permitted gradual cooling of samples to -80°C under a preset cooling profile.

#### Translational cryostage

**Fig 1:**
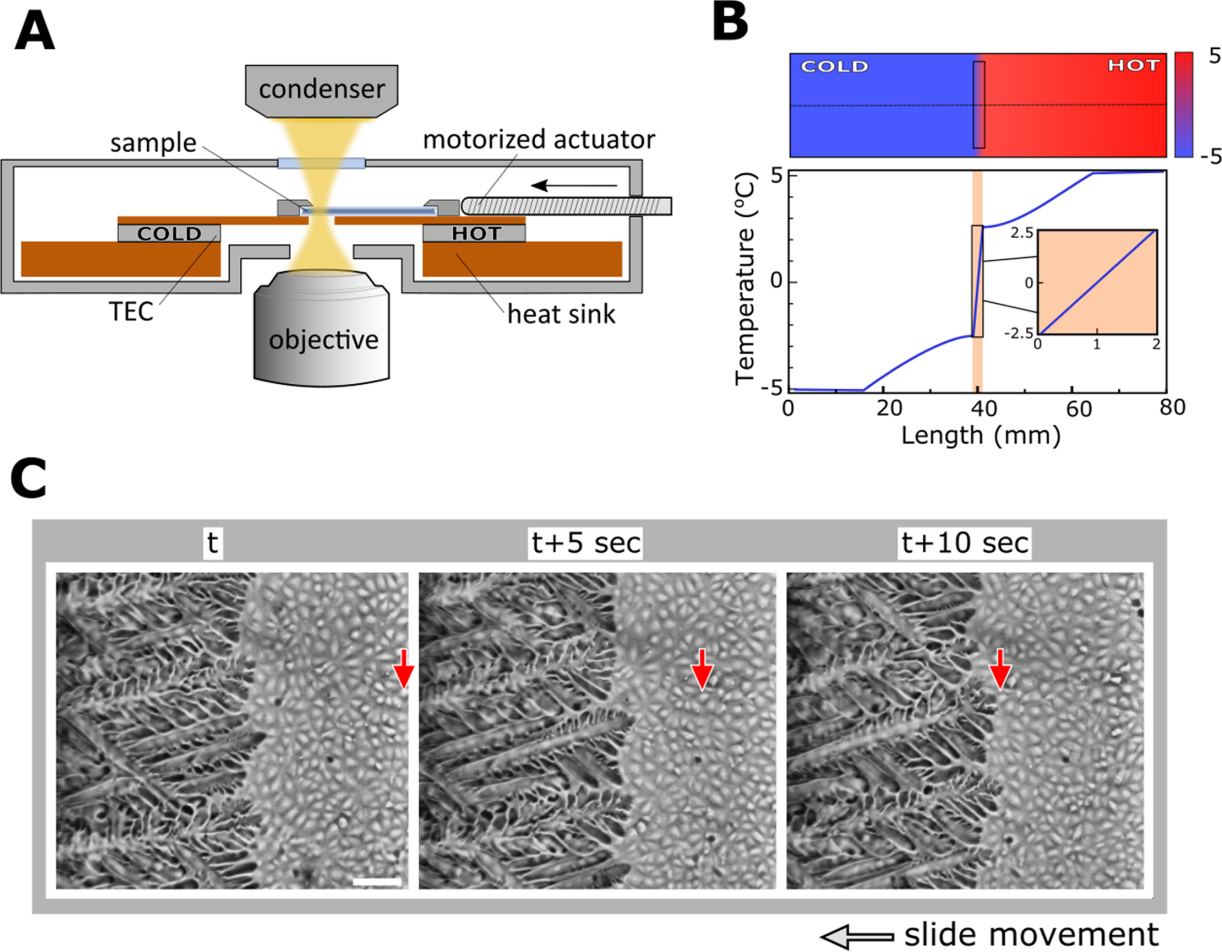
Transnational cryostage for directional freezing of cells adherent to a substrate. (A) Two independently controlled TECs controlled the temperatures on top of the 1.5 mm thick copper plate. “Cold” and “hot” bases of the thermal blocks separated by 2 mm slit. A motorized actuator enabled movement of the sample at a precise velocity. (B) Temperature field with a unidirectional gradient simulated with COMSOL Multiphysics. The temperature profile along the direction of the gradient displayed a steep linearity on top of the slit (inset). (C) Representative image sequence of a growing ice front in a 10% v/v DMSO solution. The front remained in the middle of the imaging frame while cells moved (displacement of a specific cell is indicated by a red arrow). The scale bar indicates 100 μm.

Adopting the conceptual design of a translational cryostage introduced by Rubinsky et al [29], we developed a computer controlled version of the stage. The cooling core of the stage consisted of two copper surfaces separated by a 2 mm slit (Fig 1A), wherein the surface temperatures could be independently controlled using a high-precision proportional–integral–derivative (PID) temperature controller (PRO800 system with two TED8020 modules, ThorLabs). Cooling was performed using a Peltier thermoelectric cooler (TEC) (06311-5L31-02CFL, Custom Thermoelectric, USA). The temperature was measured using a 10 kΩ Thermistor (G1540, EPCOS AG, Germany). Unidirectional freezing was achieved by setting the temperatures of the TECs above and below the melting temperature (T_h_ and T_c_) and moving the sample towards the colder side of the stage. COMSOL Multiphysics simulation was used to model the unidirectional temperature field gradient across the stage (Fig 1B). Within the slit, the temperature gradient could be expressed as:

$$\nabla T=\alpha ∙(T_{h}-T_{c})/d$$ (1)

Where *d* is the width of the slit between the copper plates and α is a prefactor that depends on the thermal conductivity and specific geometry of the slit (Fig 1A and 1B). With COMSOL simulation of the geometries and materials in our configuration we found that α ≈ 0.7.

The movement speed (*v*) of the glass slide on top of the slit was adjusted using a linear actuator (TRA25CC, Newport, USA) that enabled continuous imaging of the ice front without moving the CCD camera (DMK 23U618, The Imaging Source, Germany). Custom LabVIEW software was written to control simultaneously the sample’s position, movement speed, temperature of the individual bases and image acquisition. To avoid water condensation inside the cryostage, we purged it with cold dry air at a rate of 0.1 L/min.

The movement velocity and thermal gradients employed in this study were sufficiently low to preserve steady-state temperature distribution during sample movement [30]. Under these conditions, the velocity of the ice front propagation was determined by the velocity of the sample movement across the temperature gradient keeping the ice front within the imaging frame of the camera (Fig 1C, S1 and S2 Movies). Since conventional freezing methods are typically described in terms of the freezing rate, we define herein the freezing rate (R) as *R* = *v* ∙ ∇*T*.

#### LN flow cooling stage

An additional stage was used to gradually cool the samples to -80°C. It consisted of an aluminum block (80 mm X 80 mm X 20 mm) with a U-shape flow channel (8 mm in diameter), a 5 mm thick copper plate attached by screws to the top of the aluminum block, and a thermocouple placed on top of the copper plate (S3 Fig). The assembly was thermally isolated from the bottom and sides with PVC foam. The flow and evaporation of the LN inside the aluminum block cooled the copper plate. During cooling, a sample was placed on top of the copper plate and covered with a polycarbonate box, which was purged with dry air to avoid condensation of water onto the sample. The cooling profile was regulated using a LabVIEW PID loop feedback that controlled the flow rate of LN using a cryogenic solenoid valve (VCW31-5C-5-02N-C-Q, SMS, Japan).

### Cells freezing process

The translational cryostage was cooled below -20°C to create a frozen drop of distilled water on top of a glass slide (170 μm thick) that was positioned on top of the stage. The temperatures of the thermal blocks were set to 3°C above and below the melting temperature of the freezing medium. Confluent Caco-2, HeLa, or IEC-18 cells grown on 11 or 18 mm diameter glass cover slips in 24 or 12 well plates (Thermo Fisher Scientific—Nunc A/S, Waltham, MA, USA) respectively, were transferred to a 60 mm culture dishes (Thermo Fisher Scientific) pre-filled (2.5 mL) with the freezing medium containing 90 to 100% DMEM culture medium fully supplemented as described above and 10 to 0% v/v DMSO respectively. After 5 min incubation in the freezing medium, the coverslip covered with the cell monolayer was placed on top of the thin glass slide (cells facing down) in contact with the frozen drop to provide an initial nucleation site. For directional freezing (step (1), S1 and S2A Figs) linear movement of the coverslip was initiated on the stage towards the colder thermalblock. Three different movement velocities were tested: 10 μm/sec, 30 μm/sec, and 90 μm/sec, corresponding to cooling rates of 1.3°C/min, 3.8°C/min, and 11.3°C/min, respectively. The slowest velocity of 10 μm/sec was chosen according to the conventional cooling rate of 1°C used for cryopreservation of suspended cells. Faster velocities were applied attempting to shorten the freezing time and improve the survival rates. Upon completion of the directional freezing step, the temperature of the hot thermal block was equilibrated with the temperature of the colder thermal block. Freezing continued according to the following steps: Gradual cooling to -20°C carried out on the translational cryostage (step (2), S1 and S2A Figs) at a rate of 1.2°C/min, followed by deeper cooling to -80°C, carried out by transferring the frozen sample onto the LN flow cooling stage, which was precooled to -20°C and then decreasing the temperature gradually to -80°C at the conventional rate of 1°C/min (step (3), S1 and S2B Figs) or at a slower rate of 0.5°C/min, or alternatively, by transferring the sample onto 50 mm diameter, 4.5 mm thick copper disk pre-cooled to -20°C, before storing the frozen sample at -80°C for at least 24 h (step (4), S1A Fig) until thawing.

### Cells revival process

For thawing, the frozen samples were transferred from the -80°C freezer to a 60 mm culture dish (Thermo Fisher Scientific) pre-filled with warmed (37°C) DMEM culture medium fully supplemented as described above, followed by culturing in a 37°C humidified atmosphere containing 95% air and 5% CO_2_. Pre- and post-freezing phase contrast images of the cells were captured using the Eclipse TS100 microscope (Nikon, Tokyo, Japan) with 10x and 40x air objectives.

### Viability assay

A live/dead assay was conducted using a commercial kit (ab115347, Abcam, Cambridge, UK) according to the manufacturer’s protocol. Briefly, the cover slips covered with the cells were transferred to 12 or 24 well plates and thawed as described above. The kit solution was mixed with PBS to produce a 1X final concentration and added to the thawed cells. After 10–15 min incubation at 37°C, live/dead staining was visualized using an inverted epifluorescence microscope (Olympus IX51) with a 20x objective. LIVE: excitation 495 nm, emission: 510–550 nm, dichromatic mirror 505 nm, DEAD: excitation 510–550 nm emission >590 nm, dichromatic mirror 570 nm.

For flow cytometry analysis; the thawed cells were immediately detached from the coverslip by trypsin treatment (Biological Industries), collected, and centrifuged. The pellet was re-suspended in a 1X solution of the dye mix and incubated for 15–60 min. The samples were analyzed using a FACSAria III (Becton, Dickinson, NJ, USA). FSC vs. SSC and FITC vs. PI plots were generated from the standard flow cytometry analysis results for 5000–10000 cells per sample.

## Results

### Optimization of the freezing procedure

#### Freezing rate effect on cell morphology

**Fig 2:**
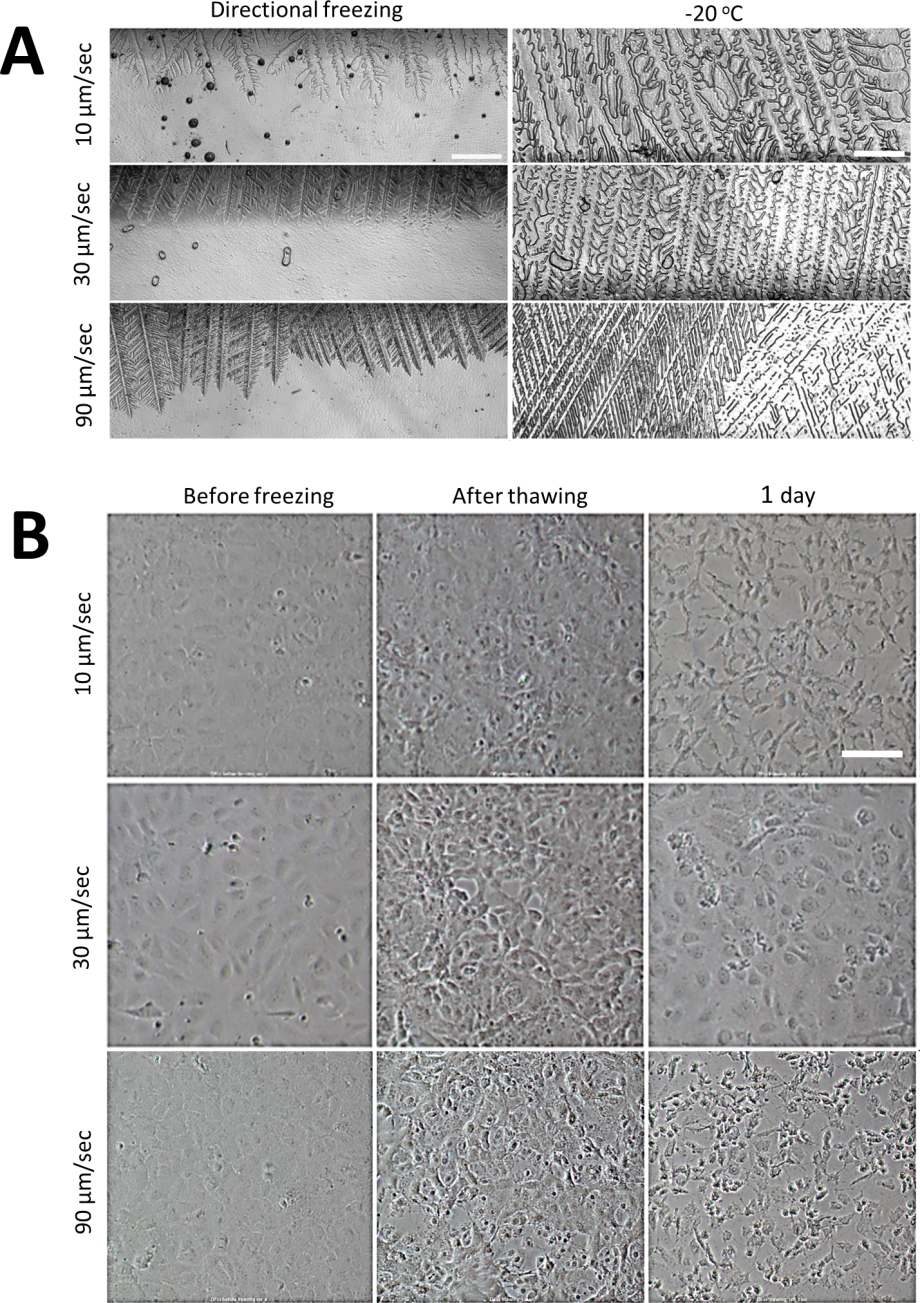
Effect of the directional freezing rate on cell morphology. IEC-18 epithelial cells cultured on a cover glass were subjected to directional cooling on the translational cryostage (10% DMSO). Sample movement velocities of 10 μm/sec (eq. 1.3°C/min), 30 μm/sec (eq. 3.8°C/min), and 90 μm/sec (eq. 11.3°C/min), were tested. Then the temperature was decreased gradually to -20°C, and the cells were transferred to -80°C. (A) Ice crystal morphology as a function of the ice front propagation. Left: during the process of freezing. Right: Frozen sample at -20°C. Scale bar indicates 400 μm. (B) Phase contrast images (10x) of IEC-18 cells prior to freezing, after thawing, and after 24 h incubation post thawing in a humidified 5% CO_2_ incubator at 37°C,. Scale bar indicates 100 μm.

We first examined the influence of the sample movement velocity, which affected both the ice crystal shape and the cooling rate. Fig 2A shows the shape of the ice crystals formed at different velocities: 10 μm/sec (eq. 1.3°C/min), 30 μm/sec (eq. 3.8°C/min) and 90 μm/sec (eq. 11.3°C/min). Increment of the velocity increased the branching of the ice crystals and decreased their width, in line with previous reports describing the directional solidification process [24, 31]. Consequently, we observed denser ice crystal texture (right panel, Fig 2A) by the end of the directional freezing step when higher movement velocities were employed.

Next, we examined the effect of the freezing rate on the morphology (as an indicator of the cell recovery/viability) of the confluent IEC-18 cell monolayers. Following directional freezing, IEC-18 cells were cooled to -20°C at a rate of 1.2°C/min on the translational cryostage. The sample was then transferred to a -80°C freezer on a copper plate precooled to -20°C. After several days (1–10 days) of storage at -80°C, the sample was thawed and imaged both immediately and after one day of culturing. Phase contrast images collected immediately after thawing (Fig 2B, "after thawing") showed a decrease in cell area and a greater contrast at the cell boundaries, suggesting osmotic shrinkage for all rates. Cells that had been exposed to faster directional freezing (90 μm/sec) displayed dark cytosol typical of intracellular freezing [32] and disrupted monolayers with multiple voids (Fig 2B, "after thawing"). Images collected one day after incubation, post-thawing, revealed that most of the cells that had been frozen at 10 μm/sec and 90 μm/sec were not viable, based on their dark and faceted morphologies (Fig 2B, "1 day"). Conversely, cells that had been frozen at 30 μm/sec appeared normal with rounded cell morphologies (Fig 2B, "1 day"). These cells also remained viable after up to a week of incubation. Remarkably, we noticed that at all freezing rates, the cells remained adhered to the substrate, even if they were not viable (Fig 2B, "1 day").

#### The effect of DMSO concentration on the cell morphology

**Fig 3:**
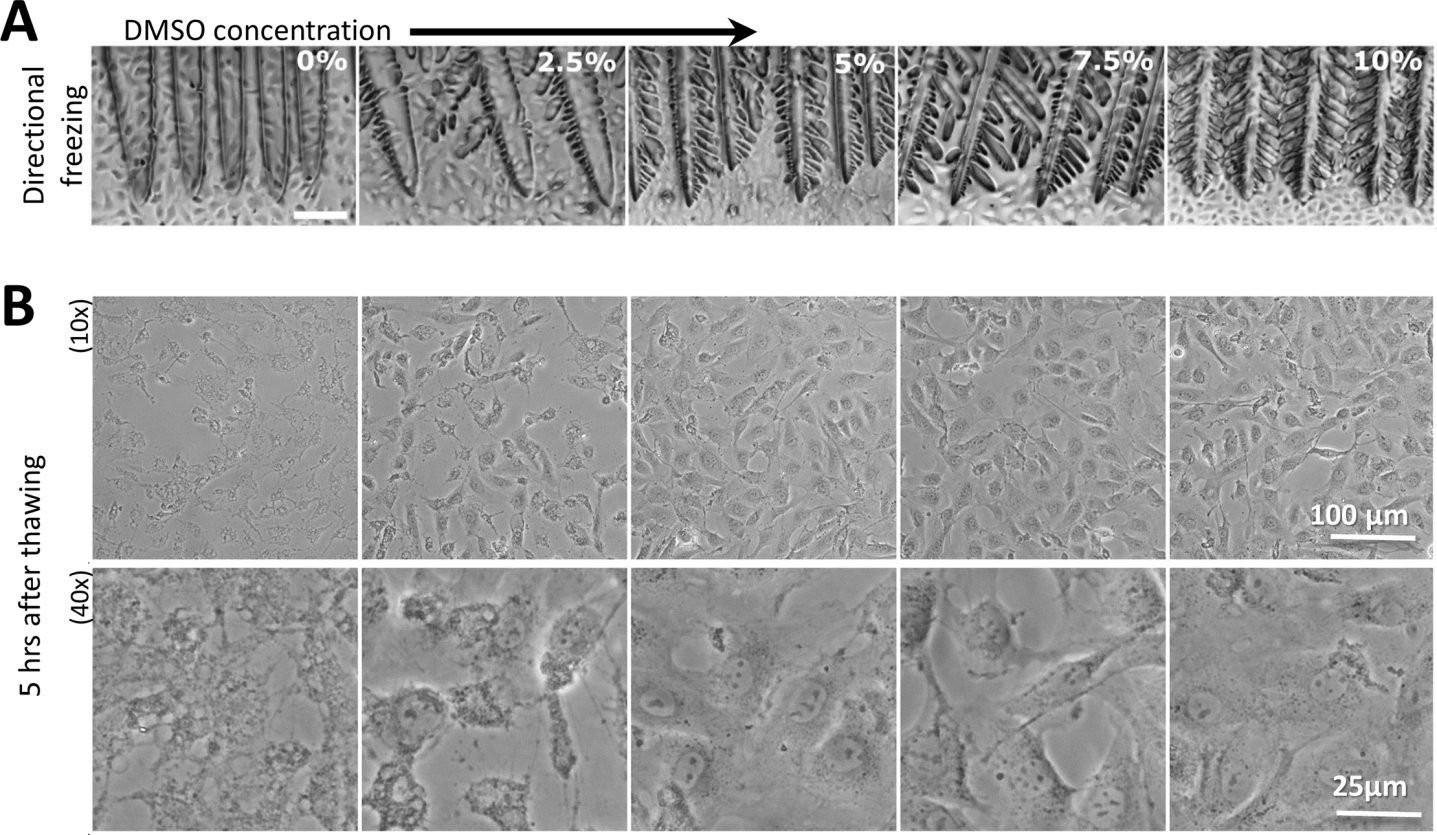
IEC-18 cell monolayer morphology after directional freezing at various concentrations of DMSO in the freezing medium. (A) Ice crystals during directional freezing (velocity = 30 μm/sec, eq. 3.8°C/min) of adhered IEC-18 cells in the presence of different concentrations of DMSO (0% - 10%). Scale bar indicates 100 μm. (B) Phase contrast images of IEC-18 cells frozen in deferent concentrations of DMSO (0% - 10%) collected after thawing and 5 h incubation in a humidified 5% CO_2_ incubator at 37°C. Scale bars indicate 100 μm for 10x and 25 μm for 40x magnification.

The effect of the DMSO concentration on cell morphology was tested by gradually reducing the concentration of DMSO in the freezing medium from 10%, typically used for IEC-18 and many other cell types, down to 0%. Higher DMSO concentrations increased the ice crystal branching instabilities, as expected for higher solute concentrations [24, 33], but the typical crystal size did not change significantly at that velocity (30 μm/sec, Fig 3A). The morphologies of the IEC-18 cells after thawing and after 5 hours incubation appeared intact, round shaped for the majority of cells preserved in 10%, 7.5%, or 5% DMSO (Fig 3B), while in 2.5% DMSO group, most cells appeared injured with only a few cells exhibiting healthy rounded morphologies. In the 0% DMSO, all cells appeared severely injured and fragmented.

#### Effect of gradual cooling rate from -20°C to -80°C

**Fig 4:**
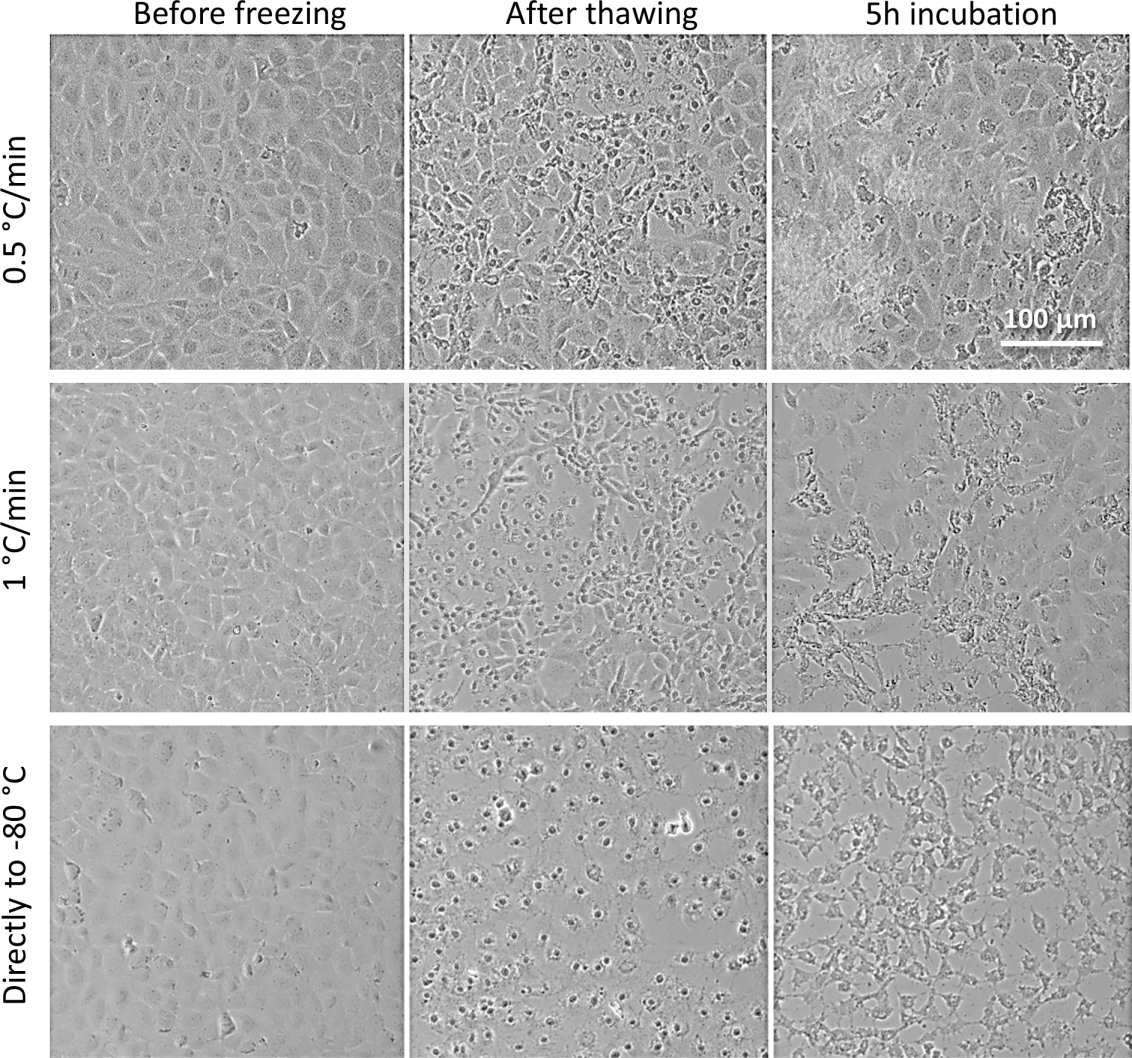
The effect of gradual freezing rate from -20°C to -80°C on cell morphology in a 10% DMSO medium. Following directional freezing of the adhered IEC-18 epithelial cells and gradual freezing on the translational stage to -20°C, samples were subjected to gradual cooling to -80°C on the LN flow cooling stage at rates of 0.5°C/min or 1°C/min. As a control, a sample was transferred directly to the -80°C freezer after reaching -20°C. Phase contrast images (10x) were collected prior to freezing, after thawing, and after 5 h incubation post thawing in a humidified 5% CO_2_ incubator at 37°C. Scale bars indicate 100 μm.

After directional freezing and gradual cooling of the cells down to -20°C on the translational cryostage, the samples were transferred to the LN flow cooling stage, and gradually cooled down to -80°C, below the glass transition temperature, (see the freezing scheme, S1 Fig). We examined the differences between the typical cooling rate of 1°C/min, a slower cooling rate of 0.5°C/min, and direct storage at -80°C. We found that both the 1°C/min and direct -80°C approaches yielded cell injury phenotypes (Fig 4) similar to that obtained in the fast directional freezing scheme at 90 μm/sec (eq. 11.3°C/min) (Fig 2B) or at low DMSO levels (0% and 2.5% DMSO, Fig 3B). The injury phenotype was characterized by intracellular freezing damage, as indicated by a dark cytosol and intercellular network disruption. Gradual cooling dramatically increased the survival of cells in comparison to direct storage at -80°C, under which conditions survival rates were practically zero (5 h, Fig 4). Lowering the cooling rate to 0.5°C/min resulted in an even higher survival rate (5 h, Fig 4). Interestingly, we observed in several cases when the cooling step was not completed due to a technical issue, that -40°C appeared to be a critical point where transferring the cells to the -80°C storage above this temperature resulted in 0% cell survival, while below this temperature cells survived at a significantly higher rate.

### Assessment of human cell lines survival by cell morphology

**Fig 5:**
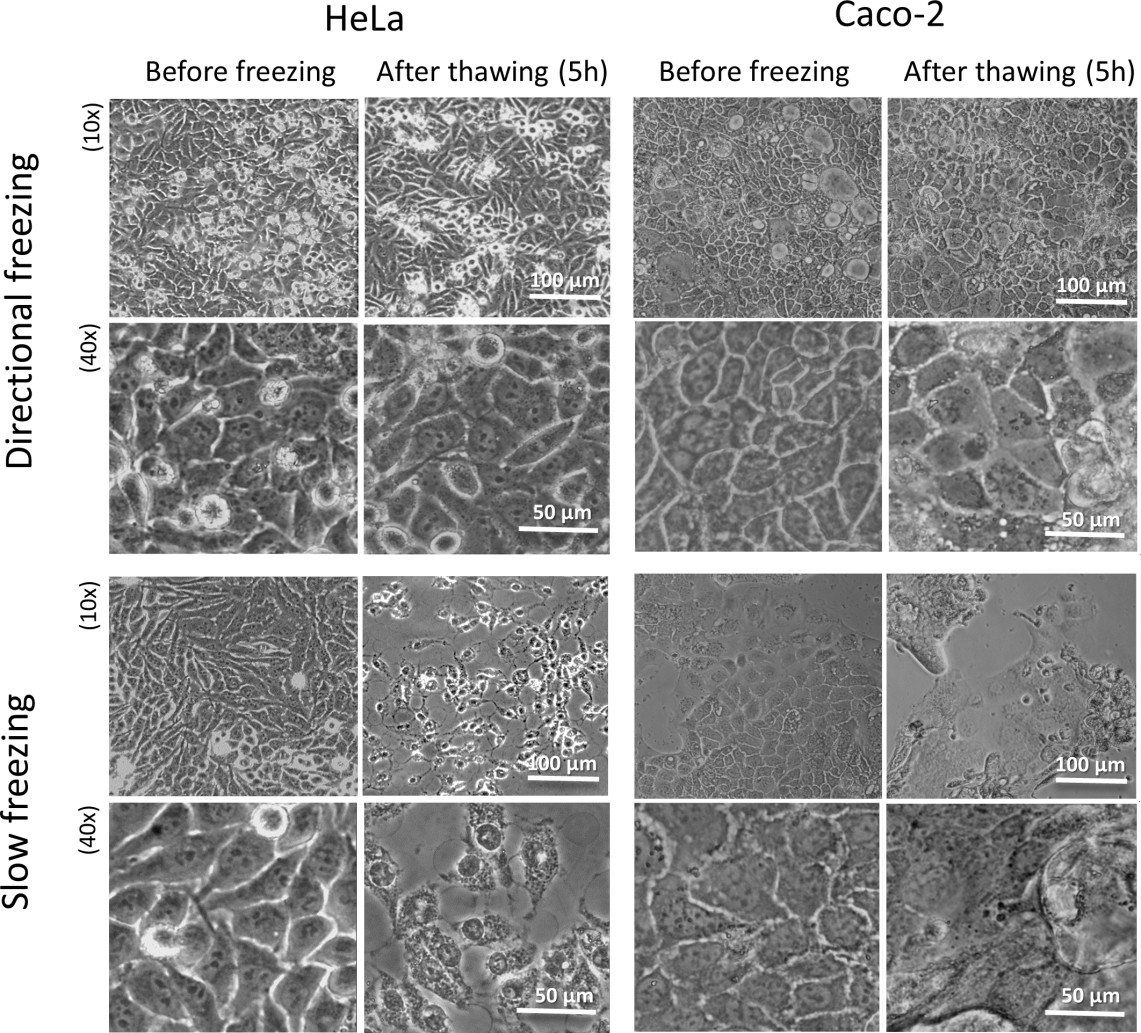
Viability of the HeLa and Caco-2 cells after directional freezing vs. non-directional slow freezing. Phase contrast images of the HeLa (left panel) and Caco-2 (right panel) cell cultures prior to freezing and 5 h after thawing. Cells frozen at 10% DMSO using a combination of directional freezing at a speed of 30 μm/sec and gradual cooling at 0.5°C/min (upper panel), or solely using gradual cooling at 0.5°C/min (lower panel). Scale bars indicate 100 μm for 10x and 50 μm for 40x magnification.

We next examined our optimized cryopreservation protocol on two ubiquitous human cell lines, HeLa and Caco-2 cells. The parameters used were: 10% DMSO freezing medium, a translational velocity of 30 μm/sec (eq. 3.8°C/min), gradual cooling at 1.2°C/min to -20°C, followed by gradual cooling at 0.5°C/min down to -80°C. This protocol was compared with non-directional gradual freezing to -80°C at a conventionally used cooling rate of 1°C/min. Bright field images of the cells (collected 5 h post thawing) clearly revealed that both HeLa and Caco-2 cell survival was significantly higher under the directional freezing approach as compared to the non-directional slow freezing (Fig 5). Samples subjected to directional freezing were characterized by intact monolayers with normal cell shapes and bright clear nuclei. On the other hand, samples that had been cryopreserved using non-directional freezing appeared with darker cells that had lost their cell–cell attachments within the monolayer. Interestingly, many of the Caco-2 cells detached from the glass slide after the thawing procedure. This did not affect the healthy appearance of the directionally frozen cells, which remained on the substrate.

#### Viability assay by fluorescence staining

The survival rates were further tested using a live/dead staining assay. The number and the ratio of live and dead cells analyzed by fluorescence imaging 5 hours after thawing and by flow cytometry where cells were collected immediately after thawing to count for cells that could detach in course of post-thawing incubation.

**Fig 6:**
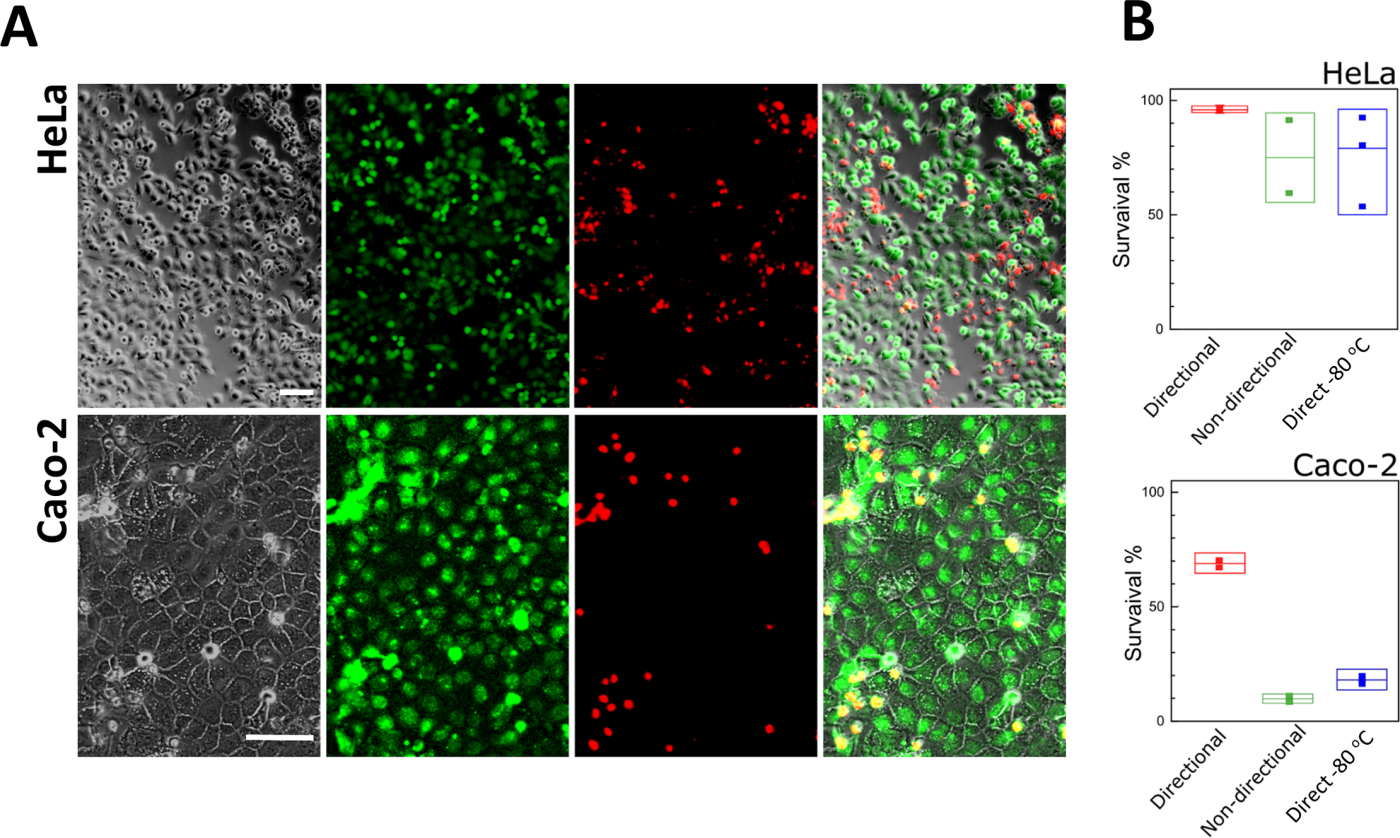
Quantification of cell viability. **(**A) Adherent HeLa and Caco-2 cells labeled 5 h after thawing using the live/dead kit (green—*live*/ red- *dead* cells). Scale bars indicate 100 μm. (B) Quantification of live/dead labeling using flow cytometry performed on cells that were collected immediately after thawing compared the cell survival percentage obtained under combination of directional freezing and gradual cooling, non-directional gradual cooling (1°C/min) and direct freezing at a -80°C freezer.

HeLa cells that underwent cryopreservation under directional freezing showed consistent very high survival rates (90–100%) after thawing, as observed by fluorescence microscopy images (Fig 6A) and flow cytometry results (Fig 6B). By contrast, other cryopreservation methods, such as non-directional slow freezing and direct storage in -80°C freezer (“direct -80°C”, Fig 6B), applied to HeLa cells showed variable results, with as low as 50% survival rate after thawing.

Similar studies of Caco-2 cells revealed even greater advantages under the directional freezing scheme, with up to 60% cell survival after thawing, compared to 20–30% using the non-directional slow freezing and direct -80°C methods (Fig 6B).

### Robustness of the optimized cryopreservation protocol

In this study, we performed over 100 independent cryopreservation experiments. 75% of the data were analyzed for survival rates based on cell morphology assessments 1 day after thawing, and the rest were removed due to technical failures during the experiments (e.g. human mistakes, power brakes, running out of LN). Applying the optimized protocol involving a 7.5–10% DMSO freezing medium (see the cooling scheme shown in S1 Fig) provided significantly higher survival rates of 88.3% over 43 experiments, whereas the non-directional slow freezing at 0.5–1°C/min yielded a very low survival rate of 14.3% over 14 experiments. We defined survival as successful when the majority of cells on the slide had a normal morphology after a day of incubation.

## Discussion

Cryopreservation methods can be classified as one of two main approaches: slow cooling or vitrification. In both methods, cells are surrounded by a freezing medium cooled below the glass transition temperature without inducing cell freezing. Vitrification, which employs flash cooling with a high concentration of CPAs, completely avoids the formation of ice in the sample. On the other hand, slow cooling uses much lower CPA concentrations permitting ice formation in the extracellular medium. These factors favor slow cooling during scaling up of cryopreservation methods applied to large tissues and organs [27]. In directional slow cooling, a sample is subjected to a temperature gradient in the direction of the sample movement which induces the controlled growth of ice crystals in the same direction, leading to the formation of uniformly distributed ice crystals throughout the sample [29, 34].

Cells cryopreservation procedures ideally hinder the formation of ice crystals inside the cells (intracellular ice) [6, 7] as the cells are cooled below the glass transition temperature. Thus, an optimal slow cooling regime has to allow water outflow from the cells, as ice volume increases in the extracellular medium. It ensures that the cells are maintained at a higher concentration of solutes compared to the extracellular medium. On the other hand slow cooling has to avoid significant dehydration of the cells due to prolonged exposure to high salt concentrations [7, 35]. The cryopreservation of cells in an adherent state in addition requires preservation of the cell–substrate [13] and cell–cell adhesion networks [3, 32].

### Directional freezing

The optimal speed of ice front solidification was obtained using a typical 10% DMSO concentration. A translational velocity of 30 μm/sec, equivalent to a cooling rate of 3.8°C/min, resulted in the most successful preservation of IEC-18 cell epithelial monolayers and post-thawing viability. In contrast to the conventional slow freezing—cooling rates (1°C/min) used for cryopreservation of suspended cells, directional freezing enabled us to increase the cooling rate by a factor of 4 during the translational step, which is important for minimizing cellular exposure to toxic CPAs. These favorable results obtained at this specific velocity and cooling rate could be explained by several factors and/or their combination: (i) a uniform ice crystal distribution produced during directional freezing enabled faster cooling of the cells without significant undercooling, which can cause intracellular freezing; (ii) the larger surface area of adherent cells, compared to the suspended spherical cells, facilitating the faster efflux of water from cell interior; (iii) faster cooling minimized the cell exposure to high levels of toxic CPA omitting intracellular ice formation. We suggest that the directional freezing step resembles induction of ice nucleation practiced to increase survival rates in embryo cryopreservation [36]. Only that directional freezing enables ice growth rate tightly controlled by the translational velocity.

### Reduction of DMSO concentration

Assessing the minimum concentration of DMSO needed for successful cryopreservation in our protocol revealed that at a translational speed of 30 μm/sec (eq. 3.8°C/min), the DMSO concentration could be reduced by at least a factor of 2, from 10% to 5%, for the IEC-18 cells (Fig 3B). By contrast, reducing the DMSO concentration to 5% was destructive for the Caco-2 cells (S4 Fig). Post-thawing results obtained from the IEC-18 cells cryopreserved at low DMSO levels (2.5%) suggested a non-viable morphology, although these cells remained attached to the substrate (Fig 3B). This fact suggests that the major cellular injury at low DMSO levels results from intracellular freezing rather than the loss of adhesion to the substrate. Furthermore, typical ice crystals did not change dramatically with the DMSO level; therefore, it is unlikely that ice crystal morphologies played an important role in cell survival here. In the case of the Caco-2 cells, most of the layer peeled away at lower DMSO concentrations, in agreement with the observation that Caco-2 cells detach from a substrate (S4 Fig) under specific signaling.

### Gradual freezing (-20°C to -80°C)

During gradual cooling to -80°C, a rate of 0.5°C/min was found to be more beneficial for cell survival than the conventional rate of 1°C/min typically used to freeze suspended cells (Fig 4 and S5 Fig). This significant decrease in the optimal cooling rate at low temperatures may be explained by longer times required for the cells to pump out water in order to maintain osmolarity equilibrium with the extracellular medium when the temperature decreases. This is in line with the fact that extracellular osmolarity is determined by the temperature according to Blagden's law, and with the fact that the DMSO solution viscosity increases exponentially as the temperature decreases [37] implying that the cooling rate should be slowed at lower temperatures to compensate for the lower water mobility.

### Cell-specific freezing protocol

In this study the survival rates of the adhered Caco-2 cells and HeLa cells were compared after directional freezing, revealing that the HeLa cells were more tolerant to freezing under these conditions than the Caco-2 cells (90% vs. 60% post thaw viability) (Figs 5 and 6). This may imply that the cryopreservation protocol for Caco-2 cells as well as for other cell types may need cell specific optimization, such as the confluency level upon freezing, the freezing solution composition, or surface activation followed by extra cellular matrix coating of the glass slide.

### The significance of cryopreservation on cellular health and prospective research

Although cryopreservation techniques are widely used, emerging data suggest that cryopreservation procedures affect cell survival, cell function, protein expression, and DNA integrity, as well as the cytoskeletal and nuclear structures [7, 38–41]. Common CPAs, such as DMSO, have been shown to display toxic effects on cells that are considered to be temperature-, time-, and concentration-dependent [39, 42, 43]. It is thus become apparent that reducing the exposure to CPAs may be critical for improving in-vitro models of disease used in mechanistic and drug development research, as well as in the emerging field of stem cell research. Several studies have shown that cryopreserving stem cells can reduce pluripotency marker expression and reduce the cells’ capacity to differentiate into specific lineages or, alternatively, to trigger spontaneous differentiation [38, 44–48]. These features may be cell type specific [49]; therefore, the development of successful cryopreservation methods that reduce the local CPA exposure of the cells, for example by applying directional freezing, is a required step for advancing a wide range of cell-based in vitro models.

## Conclusion

By combining directional freezing and gradual cooling at specific rates, we were able to present a proof-of-concept for successful cryopreservation of cells adherent to a substrate. Two types of computer controlled cooling stages, translational cryomicroscopic system and LN cooled cryostage, were developed for this goal. Different cooling regimes and freezing medium compositions were tested and optimized concluding in a new cryopreservation protocol presented herein. The protocol consists of the following key parameters: 10% DMSO, directional freezing with a translational velocity of 30 μm/sec (equivalent to cooling rate of 3.8°C/min), followed by gradual cooling at a rate of 1.2°C/min to -20°C and deeper gradual cooling at a rate of 0.5°C/min to -80°C. We found that the directional freezing phase, which facilitates formation of evenly distributed ice crystals in the extracellular medium should be carried out at faster rate than expected in cell suspension cryopreservation (1°C/min). In our interpretation this step reminiscent of the “ice seeding” practiced in embryo cryopreservation [36]. We suggest that the cooling rate in cell culture cryopreservation should be slowed down with the decrease of temperature, which is also in line with a protocol applied in embryo cryopreservation [36]. Our approach displays high reproducibility and high survival rates compared to non-directional slow freezing methods and other previously reported attempts of cryopreservation of cells in an adherent state [3, 12–18, 21]. Our method can be readily applied as a robust cryopreservation protocol for various in vitro applications. We believe that the demonstrated capacity will pave the way for cryopreservation of multicellular networks and complex tissues preserving contact between the cells and adhesion with the substrate allowing new cell banking opportunities and development of new off-the-shelf cell based assays.

## Supporting information

S1 Fig Schematic diagram showing the optimized cryopreservation procedure. (A) Step (1) Directional cooling at 30 μm/sec (eq. 3.8°C/min). Step (2) Gradual cooling on the translational cryostage down to -20°C at 1.2°C/min. Step (3) Gradual cooling to –80°C, carried on the LN flow cooling stage at a rate of 0.5°C/min. Step (4) Storage of the frozen sample at -80°C and thawing, step (5). (B) Freezing procedure, as executed by means of translational cryostage and gradual LN cooled stage. The cell sample is moved on top of temperature gradient towards the colder thermal base, step (1). Then, gradually cooled (1.2°C/min) down to -20°C on the translational stage, without being moved, step (2). Finally, the sample is placed on the LN cooling stage and gradually cooled (0.5°C/min) down to -80°C/min, step (3) for prolonged storage.
(TIF)

S2 Fig Temperature profiles during the cryopreservation procedure. (A) During directional cooling the hot (red line) and the cold (blue line) thermal bases kept at constant temperature ±0.02°C/min (inset). After directional freezing the temperature of the hot thermal base equilibrated with the cold base and initial gradual cooling down to -20°C was carried out at a rate of 1.2°C/min. (B) Deep gradual cooling on liquid nitrogen cooled stage at rates of 0.5°C/min and 1°C/min (red and blue lines respectively).
(TIF)

S3 Fig Liquid nitrogen cooled computer controlled stage. (A) Schematic illustration of the system. (B) A photograph of the cold stage.
(TIF)

S4 Fig The effect of DMSO concentrations in the cryopreservation solution on adhered Caco-2 cell morphology after directional freezing. Phase contrast images with 10x magnification (panel A) and 40x magnification (panel B) were taken before freezing, after thawing and after incubation for 5 h post thawing in humidified, 5% CO_2_ incubator at 37°C.
(TIF)

S5 Fig The effect of gradual freezing at -20°C to -80°C range on adhered HeLa cell morphology in a 10% DMSO medium. Following directional freezing and gradual freezing on the translational stage to -20°C, the samples were subjected to gradual cooling to -80°C on the LN flow cooling stage at rates of 0.5°C/min or 1°C/min. As a control, the sample was transferred directly to -80°C after getting to -20°C. Phase contrast images (10x magnification) were taken before freezing, after thawing and after 5h and 24h post thawing incubation in humidified, 5% CO_2_ incubator at 37°C.
(TIF)

S1 Movie Directional freezing of IEC-18 cell culture adhered to glass coverslip in freezing medium supplemented with 10% v/v DMSO. Translation speed 30 μm/sec corresponding to cooling rate of 3.8°C/min. Magnification 10x.
(AVI)

S2 Movie Directional freezing of IEC-18 cell culture adhered to glass coverslip in freezing medium supplemented with 10% v/v DMSO. Translation speed 30 μm/sec corresponding to cooling rate of 3.8°C/min. Magnification 20x.
(AVI)
