# Supplementary figures and images for: Directional freezing for the cryopreservation of adherent mammalian cells on a substrate
Source: PLoS One. 2018 Feb 15;13(2):e0192265. doi: 10.1371/journal.pone.0192265 (PMC5813933; doi:10.1371/journal.pone.0192265)

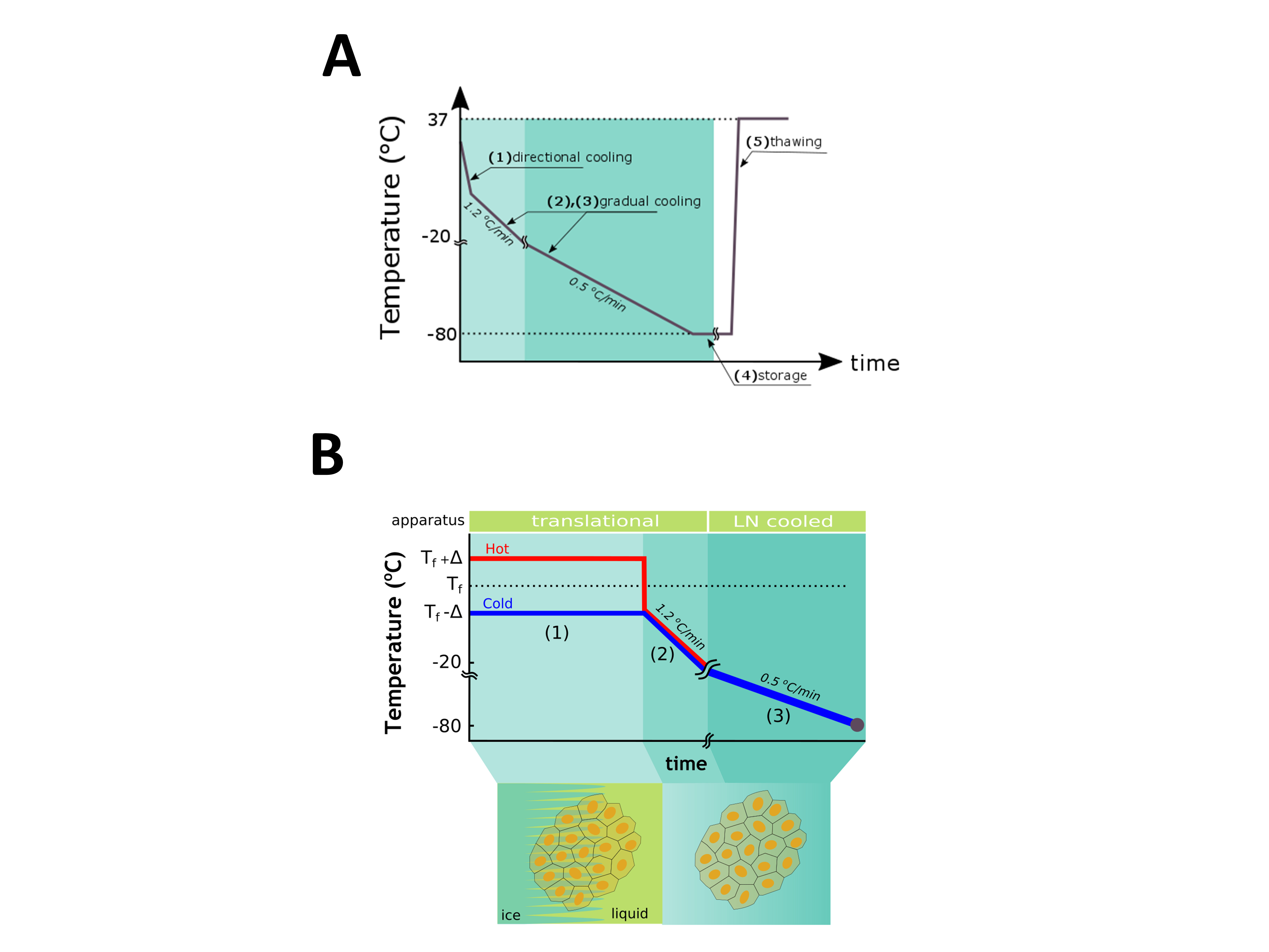

Supplement: S1 Fig — (A) Step (1) Directional cooling at 30 μm/sec (eq. 3.8°C/min). Step (2) Gradual cooling on the translational cryostage down to -20°C at 1.2°C/min. Step (3) Gradual cooling to –80°C, carried on the LN flow cooling stage at a rate of 0.5°C/min. Step (4) Storage of the frozen sample at -80°C and thawing, step (5). (B) Freezing procedure, as executed by means of translational cryostage and gradual LN cooled stage. The cell sample is moved on top of temperature gradient towards the colder thermal base, step (1). Then, gradually cooled (1.2°C/min) down to -20°C on the translational stage, without being moved, step (2). Finally, the sample is placed on the LN cooling stage and gradually cooled (0.5°C/min) down to -80°C/min, step (3) for prolonged storage. (TIF) [file pone.0192265.s001.TIF]

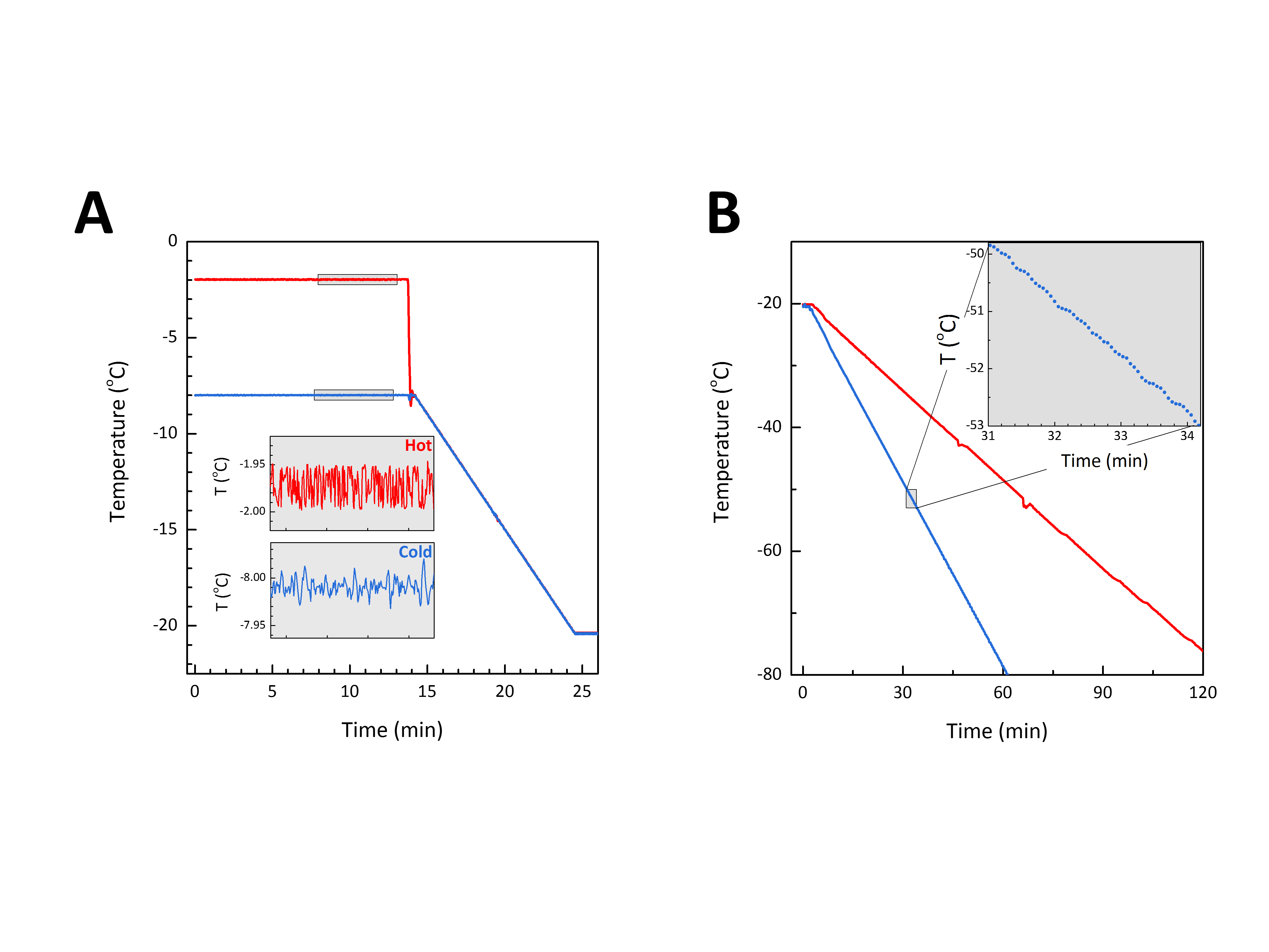

Supplement: S2 Fig — (A) During directional cooling the hot (red line) and the cold (blue line) thermal bases kept at constant temperature ±0.02°C/min (inset). After directional freezing the temperature of the hot thermal base equilibrated with the cold base and initial gradual cooling down to -20°C was carried out at a rate of 1.2°C/min. (B) Deep gradual cooling on liquid nitrogen cooled stage at rates of 0.5°C/min and 1°C/min (red and blue lines respectively). (TIF) [file pone.0192265.s002.TIF]

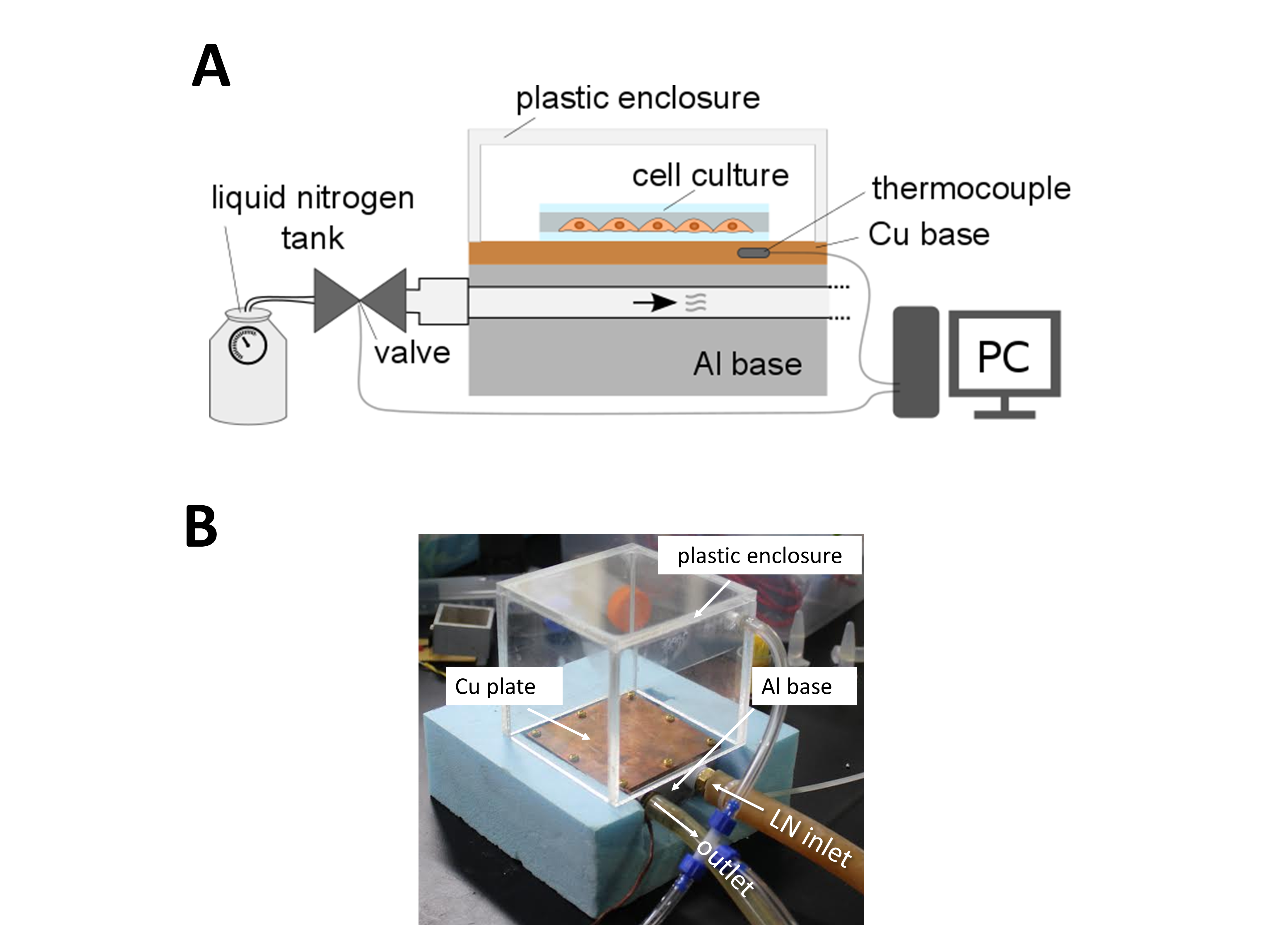

Supplement: S3 Fig — (A) Schematic illustration of the system. (B) A photograph of the cold stage. (TIF) [file pone.0192265.s003.TIF]

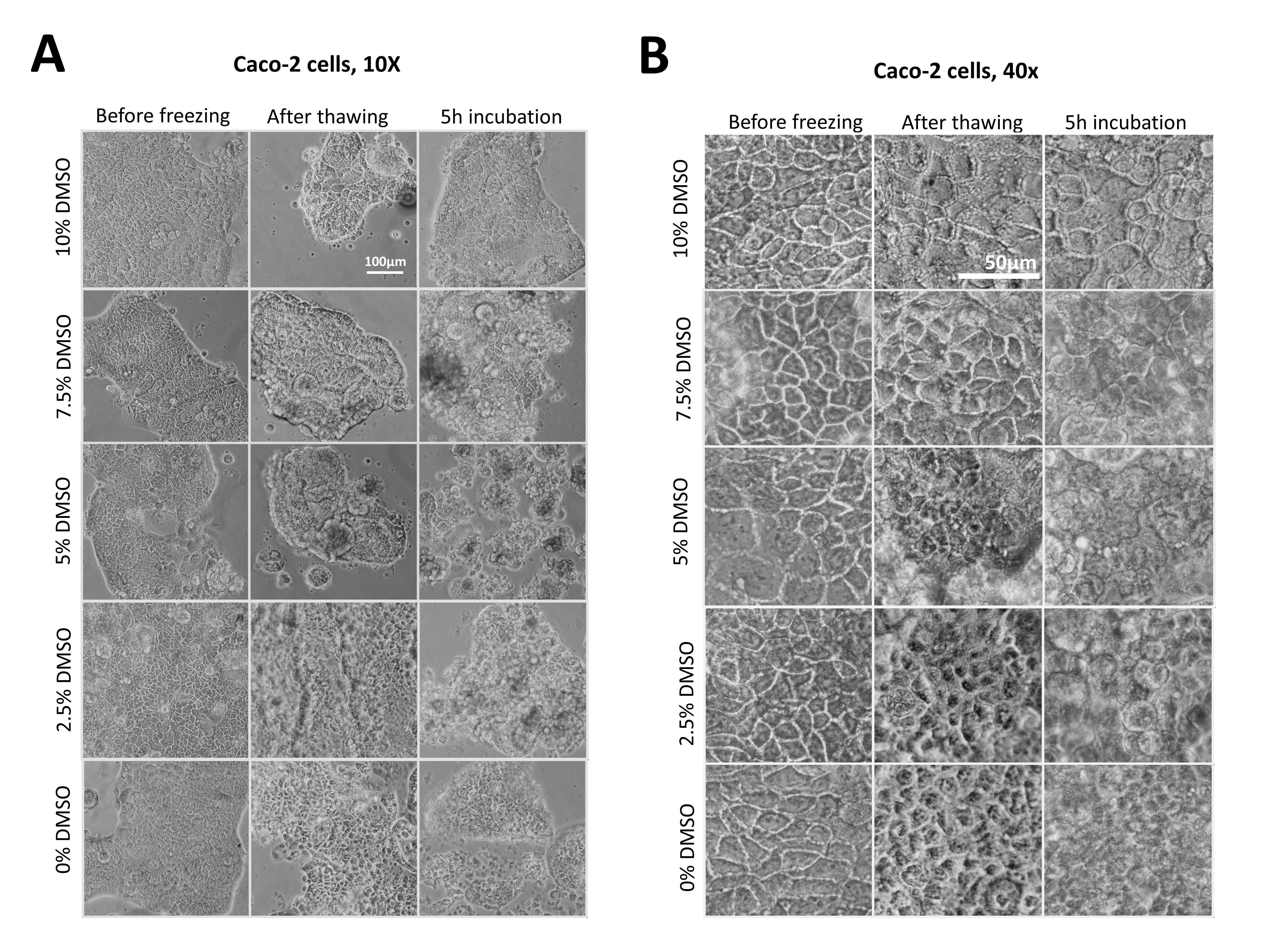

Supplement: S4 Fig — Phase contrast images with 10x magnification (panel A) and 40x magnification (panel B) were taken before freezing, after thawing and after incubation for 5 h post thawing in humidified, 5% CO2 incubator at 37°C. (TIF) [file pone.0192265.s004.tif]

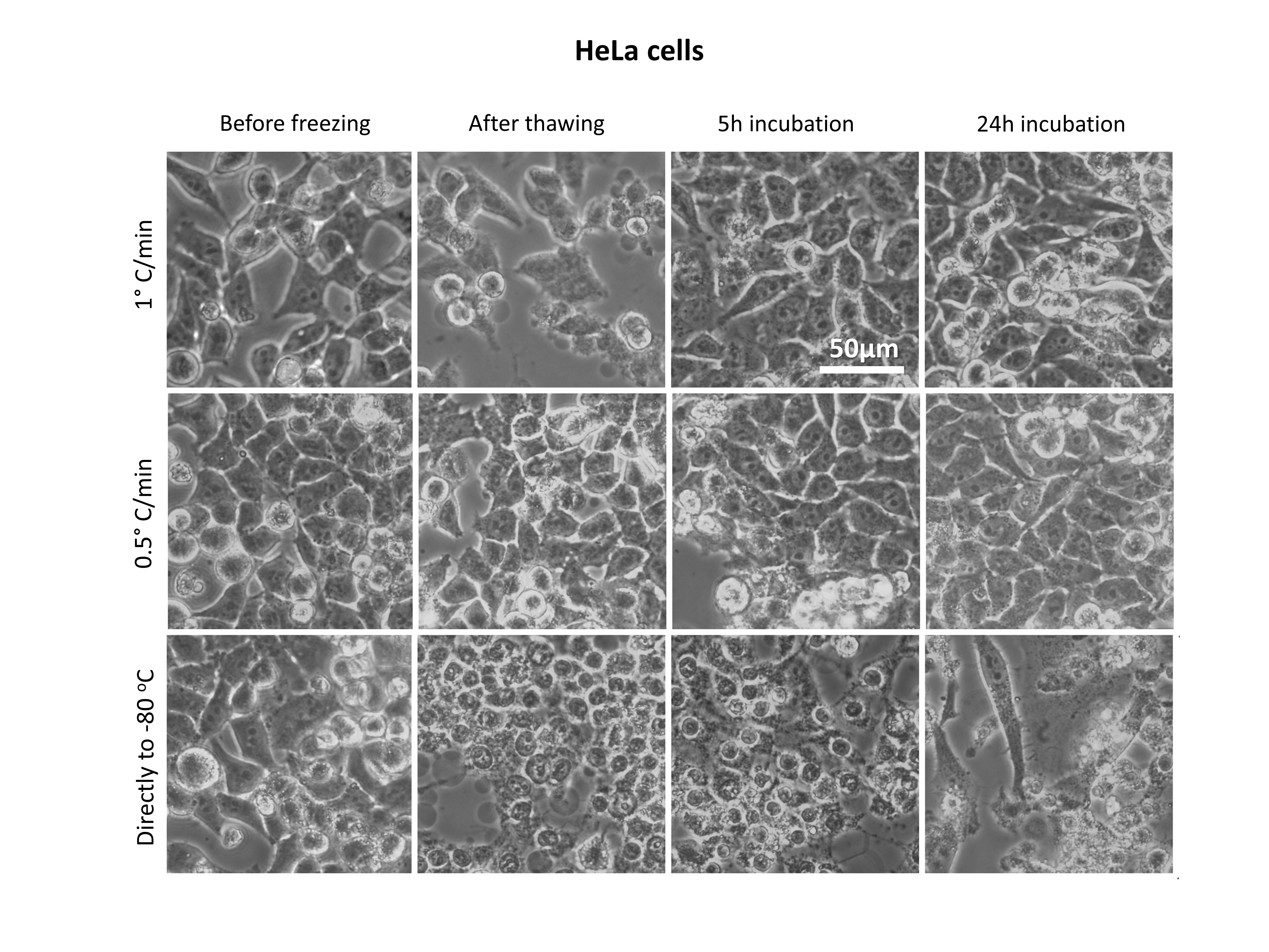

Supplement: S5 Fig — Following directional freezing and gradual freezing on the translational stage to -20°C, the samples were subjected to gradual cooling to -80°C on the LN flow cooling stage at rates of 0.5°C/min or 1°C/min. As a control, the sample was transferred directly to -80°C after getting to -20°C. Phase contrast images (10x magnification) were taken before freezing, after thawing and after 5h and 24h post thawing incubation in humidified, 5% CO2 incubator at 37°C. (TIF) [file pone.0192265.s005.TIF]
